# Round the Hospitals

**Published:** 1918-08-17

**Authors:** 


### ROUND THE HOSPITALS.

IN the distribution of honours and decorations the advisers of the King have to steer a close course between making so many recommendations that all sense of distinction in the recipient is lost, and overlooking many with a high claim to honourable mention. It was clear from the very outset that they could not please everybody, but personally we have never heard a murmur from any who have been overlooked. A correspondent, while expressing satisfaction at the awards bestowed on nurses for heroism when the hospitals in which they were serving were bombed by the enemy, puts in a claim for the special recognition of nurses serving on hospital ships torpedoed by the enemy. "Surely," he writes, "the noble heroism of the nurses on the *Gloucester City*, the *Warilda*, and other vessels, nurses who sustained the peril and ordeal of transferring their patients to the boats from a sinking steamer at night, and faced with coolness and bravery all the dangers of shipwreck with no thought for themselves, but only for the safety of their patients, should be deemed equally deserving of decorations and distinctions"? In this we are entirely at one with our correspondent, and we confidently expect that before long he will witness the accomplishment

**ROUND THE HOSPITALS—(*continued*).**

of his desires. After the recent sinking of the hospital ship *Warilda*, having on board about six hundred wounded, in addition to the crew and the medical and nursing staff, more details were allowed to transpire than ever before relating to the transport of the cot cases to the boats in pitch darkness, and all who read these accounts must have shared the wish that not a single one of those who assisted in these deeds should be overlooked.

We have often had occasion to point out the value to this country of studying methods of maternity and child welfare work in the United States and in the Dominions. It is one of the objects of endowing nursing scholarships that these methods should be studied in their proper environment. Now, through the generosity of the American Red Cross Society, the opportunity of widening the range of experience in maternity welfare is to be brought within reach of all interested in its development. The American Red Cross has actually allocated £5,000 to the National League for Health, Maternity, and Child Welfare, for the purpose of establishing and maintaining infant welfare institutions for a year. More than that, it has bestowed on this body £10,000 to found maternity hostels. There are to be U.S. hostels in London, in England, Scotland, Ireland, and Wales, for the reception of lying-in mothers, and ante-natal clinics, factory crèches, and nurseries for children of the professional classes whose mothers are at work. It may come as a shock to learn that we are considered by our sisters across the seas to be in urgent need of succour for our lying-in mothers and infants. But the sooner the nation opens its eyes to see itself as others see it the greater hope there will be of that wider spirit of municipal enterprise, which can alone adequately alter the whole situation.

Meantime what the Americans are helping us to do in this country, we and they are continuing to do in Jerusalem. Very inspiring news comes through from a correspondent of the *Times* of the altered conditions of affairs in that town and district since the Allies took it over from the Turks. The foundation of sanitation has been laid in the provision of a good water supply, and already practical immunity from cholera and typhus has been secured. Side by side with the measures which have brought about these results, a movement is on foot for the relief of suffering motherhood and the rescue of the babies. An infants' welfare bureau has been founded, where mothers are seen before and after childbirth, and a body of health visitors is being formed. While there are trained nurses, British and American, at work under the medical staff of the Occupied Enemy Territory Administration, there are also many local nurses, trained as far as circumstances admit, and invaluable in the homes of the poor. The relief measures provide for feeding infants and the disabled poor. Already infant life, so cheap in Eastern countries, is rising in value. We look to the time when Jerusalem will be the great centre of training and experimentation in this direction, radiating help and new wisdom to the mothers of Palestine.

We have the satisfaction to announce that it has been decided to hold an important meeting of all members of the College of Nursing in Northumberland and Durham for the purpose of considering how best to form a local centre in Newcastle-upon-Tyne. We are therefore requested to make it known through The Hospital that all such members of the College of Nursing are hereby invited to attend this meeting at the Royal Victoria Infirmary, Queen Victoria Road, Newcastle-upon-Tyne, on Friday, August 30 next, at 3 p.m. Miss E. C. Brown, R.R.C., matron of the Royal Victoria Infirmary, Newcastle-upon-Tyne, will be glad if all those members of the College who intend to be present at the meeting on the 30th instant will send in their names to her at the infirmary not later than Tuesday, August 27, next. Newcastle is a very important city, and its Royal Victoria Infirmary one of the most up-to-date, well-organised, administered, and interesting in the country. It is fair to assume, therefore, that every member of the College of Nursing in Northumberland and Durham will make a great effort to be present at the meeting on August 30 next at 3 p.m., and vote with both hands for the latest of local centres for the College. Miss E. C. Brown is a real worker, with a good grip, and the College and its members are indeed fortunate in being called together under her leadership; for that promises not only success, but a bumper welcome for the latest College centre.

There is no suggestion of panic in the warnings which begin to be sounded in several quarters of the danger that cholera may again visit our shores. As the natural follower of war and famine, it is already rampant in Russia, and many more centres of distribution for the germ have already been formed, in Europe, than the public guesses. The means of grappling with the disease are well understood by the health authorities, but so long is it since it appeared in England that, except such as have had experience abroad, there can be few, if any, nurses qualified to recognise cases of this nature, which may occur in the homes of the poor. In the not unlikely event of isolated cases springing up in different parts of the country it would be of the utmost importance that district and village nurses should be able to recognise the early symptoms and report without delay to the proper authorities. They certainly ought to be taught the necessary precautions to be observed in dealing with such cases. The County Nursing Associations could do much to strengthen the hands of the health authorities by arranging for a few simple lectures on cholera nursing to be given in centres within easy reach of all the affiliated nurses. District nurses in coast towns particularly ought to be forearmed.

**(Continued on page 438.)**

**ROUND THE HOSPITALS—(*continued*).**

The action of some of the greater voluntary hospitals in reducing their term of training from four to three years for V.A.D. nurses who have spent two consecutive years in military hospitals has moved the Surrey Branch of the British Red Cross Society to press for the adoption of this principle by other hospital managers. When, however, the plea is made that it should be universally adopted by civil hospitals in Great Britain and Ireland, the petitioners forget that only hospitals which give a four years' training can legitimately shorten their course in favour of the V.A.D. The principle that three consecutive years of actual training, as distinguished from mere experience, is essential for the making of a nurse is the basis on which the modern profession of nursing is founded. It would be but a poor return for the patriotic services rendered by the V.A.D. workers to tempt them to enter the profession by a back-door, as it were, which could never introduce them to the highest posts in the profession. There is no lack of training-schools offering a four-year course, and we look with some confidence to see most of them offering to receive properly qualified V.A.D. nurses for a term of three years only in consideration of their past services.

The National Health Society has issued a prospectus of considerable interest to the heads of training-schools for the special training courses commencing in September for women desiring to qualify as health visitors. The courses are arranged to occupy six months, and are timed to suit the needs of students who desire to enter in January for the certificate of the Sanitary Inspectors' Examination Board, and in May for the diploma of the National Health Society. Courses of lectures on chemistry and physics in relation to food, water, soil, air, and ventilation, on statistical methods, and on municipal hygiene are designed to prepare students for either or both of these examinations. The course designed for health visitors comprises, in addition, lectures on elementary anatomy and physiology, on infectious diseases, on tuberculosis and its prevention, and on sociology, with special lectures on venereal diseases, first aid, nursing, ante-natal maternity, and infant welfare. The sanitary inspector's course includes a course on sanitary law, and on building construction and reading of plans, with visits to places of public health interest.

All who have had practical experience in training women to be nurses must, we think, be struck with the short period allowed for the assimilation of this vast mass of detailed knowledge. Quick brains may possibly in six months cram up enough to make a passable impression in the examination-room, but far, far more is required for the training of a health visitor qualified to influence others. The courses are, however, admirably suited to nurses on completion of their third or fourth year in hospital, and matrons would do wisely to call the attention of the newly certificated to the opportunity thus offered of preparing for public work. Much of the field covered has already been traversed by the trained nurse, who yet needs the final few months of intensive study before examination. The courses should also prove attractive to nurses who have done two or three years of war work and feel the call to aid in the national reconstruction. Lastly, they open up a good career to nurses who have been trained in small or special hospitals and find themselves disqualified from the higher nursing posts through no fault of their own. Without some previous hospital training students, in our judgment, are but imperfectly equipped for the duties of health visitor, even when they have gained the diploma of the National Health Society.

The Metropolitan Asylums Board are still questing for probationers, but are not succeeding too well in competing with the many Women's Corps now in need of recruits. The shortage of labour in these hospitals is so great that many wards have been closed and many serious cases refused, including hundreds of discharged Army men. We do not swerve from our conviction that the authorities are not setting to work in the right way to secure probationers. They should develop their needs locally, and the first step to success is an attractive leaflet setting forth the conditions of service, its material attractions, and its moral claims on the community. We should welcome leaflets on this branch of the Nursing Service from any of our contributors, and hope there may be some forthcoming in the competition of which we have given notice.

The conditions are as follows: 1. The leaflets must be short, from 1,000 to 2,000 words. 2. Leaflets should each deal with one branch, and one only, of nursing work, as, for instance, private nursing, fever nursing, village nursing, midwifery, and so on. Competitors may send in one or more leaflets. 3. Leaflets should be written for outsiders, and explain in easy language the conditions of training and future prospects of women who qualify for the branch of nursing selected, with the moral and material advantages of the life. 4. Intending competitors must send their names and addresses to the Editor of The Hospital, 28 Southampton Street, Strand, W.C. 2, by September 1; the leaflets must reach him at the same address not later than Saturday, September 28, 1918. 5. Provided that thirty competitors enter and thirty leaflets at least are received, three prizes will be given of £3 3s., £2 2s., and £1 1s. We shall welcome 100 competitors at least. We hope our readers will freely compete and so help the Editor, whilst they are thus helping themselves, to help the profession too.

☛ **It is our invariable practice to pay for all items of news which we may publish. The minimum payment is 5s. Paragraphs of about twenty lines in length—that is to say, of 150 words or less—are preferred. Contributions should be addressed to The Editor, The Hospital, 28 and 29 Southampton Street, Strand, London, W.C. 2, and, to be in time for the current week's issue, should reach him by the first post on Mondays.**

